# Fuzzy logic modelling of the pollution pattern of potentially toxic elements and naturally occurring radionuclide materials in quarry sites in Ogun State, Nigeria

**DOI:** 10.1007/s10653-025-02359-2

**Published:** 2025-01-27

**Authors:** David O. Jegede, T. Adeniyi Afolabi, Foluso O. Agunbiade, Olatunde S. Oladeji, Muideen R. Gbadamosi, Samuel O. Sojinu, Oluseyi Z. Ojekunle, Pakorn Varanusupakul

**Affiliations:** 1 Department of Basic Sciences (Chemistry Unit), Babcock University, Ilishan-Remo, Nigeria; 2 Division of Environmental Health Sciences, College of Public Health, The Ohio State University, Columbus, OH 43210 USA; 3 Department of Chemistry, Federal University of Agriculture, Abeokuta, Nigeria; 4 Department of Chemistry, University of Lagos, Akoka, Nigeria; 5 Department of Industrial Chemistry, Faculty of Natural and Applied Sciences, First Technical University, Ibadan, Nigeria; 6 Department of Chemical Sciences, Tai Solarin University of Education, Ijagun, Ijebu-Ode, Nigeria; 7 School of Geography Earth and Environmental Science, University of Birmingham, Birmingham, UK; 8 Department of Environmental Management and Toxicology, Federal University of Agriculture, Abeokuta, Nigeria; 9 Department of Chemistry, Chulalongkorn University, Bangkok, Thailand

**Keywords:** Fuzzy logic, Quarry, Potential toxic elements, NORMs, Ogun state

## Abstract

The accumulation pattern of some inorganic pollutants in quarry sites around Ogun State was modeled using a Fuzzy comprehensive assessment (FCA). Potentially toxic elements (PTEs) and naturally occurring radionuclides materials (NORMs) were assessed from soil samples collected from ten quarry sites in three districts (Odeda, Ajebo, and Ijebu Ode) in Ogun State. Three (3) NORMs (^40^ K, ^238^U, ^and 232^Th) were assessed using gamma spectrometer with a NaI detector while ten (10) PTEs (As, Cd, Co, Cr, Cu, Fe, Mn, Ni, Pb, and Zn) were determined by digestion method using Inductively coupled plasma optical emission spectrophotometer. The FCA was used to evaluate soil contamination, and SPSS version 21.0 was used for statistical analysis. Concentration range of PTEs(mg/kg) and NORMs(Bq/kg) in all the site are: As (5.62 ± 0.85 – 15.93 ± 2.40), Cd (BDL—1.26 ± 0.60), Co (5.56 ± 1.34 – 27.25 ± 1.14), Cr (18.68 ± 1.54 -61.43 ± 6.33), Cu (12.40 ± 1.31—82.43 ± 7.94), Fe (15,035.00 ± 81.12 – 36,520.00 ± 292.20), Mn (168.97 ± 5.93 – 353.30 ± 20.84), Ni (5.63 ± 1.99- 25.54 ± 2.50),),Pb (4.44 ± 0.8 – 17.87 ± 2.80) and Zn (42.97 ± 413 – 147.00 ± 7.50); ^40^ K (76.78 ± 44.76—2647.88 ± 179.44), ^238^U (3.24 ± 1.82—55.42 ± 24.88),and ^232^Th (5.24 ± 3.90—244.36 ± 89.84). The results were modeled into a membership function matrix of three pollution classes. The FCA of NORMs revealed that 30, 10, and 60% of the sites were pristine, moderately polluted, and heavily polluted.

In comparison, the FCA of PTEs confirmed 100% of the sites to be heavily polluted due to the accumulative effect of the PTEs. A high percentage of membership in the extremely impacted class is linked to a high concentration of Fe in all the sites due to the soil's geological structure and natural activities. At the same time, ^40^ K and ^238^U have high-impact membership in all the quarry sites. Based on the findings, there is a need for stringent pollution control measures, targeted monitoring of PTEs and NORMs and the development of region-specific environmental regulations to protect both public health and ecosystems.

## Introduction

One of the major environmental problems in the globe, even in affluent nations, is the global phenomena of rock quarrying and stone crushing. In the process of extracting rocks, overburden is removed, stones are drilled and cut, and occasionally rocks are blasted and crushed (Waweru et al., 2018). The ecology and the socioeconomic well-being of the locals in the vicinity of quarried places are impacted by the quarry’s operations and the landscape scars left by these activities (Bamgbose et al., 2014; Misthos et al., 2018). One of the main human activity that modifies the environment’s landscape is quarrying (Flavenot et al., 2014). Quarrying operations have caused a substantial and irreversible modification of the ecosystems and biological linkages (Waweru et al., 2018). Heavy metals are a significant part of PTEs and are metals with atomic numbers greater than 20, except the group 1A metal and the group 2A metal. They are metallic compounds that occur naturally and have a density greater than 5 g/cm^3^ (Singh et al., 2018). They are one of the major persistent pollutants in the environment. According to Edelstein and Ben-Hur (2018), PTEs are a class of metals and metalloids having an atomic density greater than 4000 kg/m3. According to reports, the majority of PTEs are hazardous to humans at low metal ion concentrations (Bibaj et al., 2019). PTEs are released into the soil as a result of exposure to fertilizers, sewage sludge, industrial wastewater, weathering of soil minerals, and the use of treated wastewater for land applications (Liu et al., 2018).

Quarrying activities in Ogun State are also important because of its positive impact and economic development of the country being a source of construction materials, revenue for the government through taxation and royalties and employment especially of the rural population (Divya et al., 2012). There is significant alteration of the ecosystems and ecological relationships that are irreversible because of quarrying activities (Waweru et al., 2018).

Decision science, soft and pattern recognition computing give insight into better ways of making decisions. Fuzzy set theory is a science that has been helpful in pattern recognition, making decisions on predicting the importance of the future environments under each alternative, and giving environmental clearance to the projects. Fuzzy logic is not classical mathematics but a mathematical discipline based on fuzzy set theory (Jasutkar & Khan, 2015). Zadeh (1965, 1973) proposed a fuzzy methodology to solve issues of uncertainty and vagueness, which has been applied in different fields of science and technology. Mohanta et al. (2020) confirmed fuzzy modeling as a reliable linguistic reporting method understandable to decision-makers, managers, the public, and non-experts. There are no sharp boundaries in fuzzy sets. However, a variable that varies between 0 and 1 is a true value and is defined with a membership function confirming their transformation from being a member to not being (Theodoridou et al., 2017). In the past few years, several researchers have developed and used simple fuzzy modeling to assess environmental pollutants by integrating human health risk assessment (Ilbahar et al., 2018; Mohamed et al., 2019).

Fuzzy logic has been used and applied in various environmental fields such as Fluoride risk in groundwater for drinking health(Thabrez et al., 2023) ecosystem sustainability evaluation (Jasutkar & Khan, 2015), Metal classification, pollution impact, fish production(Akintola et al., 2024), health of agricultural land and crop classification (Murmu & Biswas, 2015), assessment of pollutants in soil and sediments (Gruijter et al., 2011; Olu-Owolabi et al., 2012), river water quality evaluation (William, 2006), environmental impact and risk assessment (Garrido & Requena, 2015), bioaccumulation pattern of metals in plant(Agunbiade et al., 2012; Olu-Owolabi et al., 2013), water quality evaluation status (Nageshwaran, 2015), quality index assessment of river water (Clair & Sinha, 2015), environmental quality index, landfill siting and agricultural soils quality index (Gupta et al., 2015; Rodríguez et al., 2016). Saleh et al., (2020) highlighted the relevance of fuzzy logic in addressing the complex and multi-dimensional challenge of landfill site selection by quantifying and analyzing criteria with inherent uncertainties. By integrating fuzzy methods with analytic network process and GIS, the study achieves precise weighting and spatial analysis, enabling objective decision-making. Fuzzy logic enhances the reliability of results by accommodating imprecise data and providing flexible modeling capabilities for environmental, economic, and sociocultural criteria. The approach demonstrates its superiority over traditional methods by delivering accurate, practical solutions with minimal environmental impact, emphasizing its applicability to similar challenges in municipal waste management (Saleh et al., 2020). The listed kinds of literature mainly confirmed the use of fuzzy logic and fuzzy inference systems as a tool for decision-making for the assessment of components of environment and environmental impact. Mohanta et al. (2020) used a fuzzy-analytical procedure instead of a conventional technique to evaluate the human health risk assessment of fluoride-rich groundwater in a hamlet in India. They were able to recommend the fuzzy logic-based index method as a thorough instrument for evaluating drinking water risks related to human health. Seyed et al. (2017) have attempted to validate the effective utilization of coal fly ash-loaded synthetic hydrous iron oxide/aluminum hydroxide composite as a sorbent for Cr(VI) sorption from aqueous solution. With a correlation coefficient (R^2^) of 0.95, the fuzzy logic model performed well, demonstrating its efficacy in forecasting the efficiency of Cr (VI) removal. Moon et al. (2024) showed the relevance of fuzzy logic, specifically the fuzzy-AHP technique, in prioritizing and managing risks during nuclear decommissioning. Fuzzy logic is particularly suited for this context as it addresses the uncertainties and subjectivity inherent in risk assessment, allowing for more nuanced and flexible evaluation. By analyzing risks across tasks and identifying critical concerns such as collisions, jamming, and falling objects, the fuzzy-AHP approach facilitates comprehensive and targeted safety measures. Their research demonstrated the value of fuzzy logic in developing standardized safety protocols, enhancing decision-making, and ensuring operational efficiency in the high-stakes environment of nuclear decommissioning. Additionally, Yesilkanat et al. (2017) demonstrated the relevance of fuzzy logic in spatial risk dispersion analysis by providing a flexible and accurate method for estimating ambient gamma dose rates. By comparing fuzzy logic to various artificial neural network structures, the study highlighted its effectiveness in generating dose rate risk maps, showing comparable performance to advanced ANN models. The integration of fuzzy logic with spatial mapping ensures precise interpolation and visualization of gamma dose distribution, contributing to improved environmental monitoring and risk assessment.

Kotti et al. (2013) used a fuzzy inference systems method for wastewater treatment. They designed a model based on fuzzy logic for biochemical oxygen demand removal from wastewater, which was confirmed to be effective. Fuzzy logic has been confirmed to be an essential tool when assessing environmental risk because of its ability to use membership levels, instead of binary logic, to tackle the uncertainty and haziness of data realistically (Coutinho et al., 2015). More information is needed on the application of fuzzy logic in pollution pattern recognition in quarry mining activities and on NORMs. Fuzzy logic is particularly suited for assessing pollution because it enhances the interpretation of regulatory limits and exposure risks. Unlike traditional methods that strictly categorize values as pristine or polluted based on rigid thresholds (e.g., 2.00 ppm), fuzzy logic accounts for boundary uncertainties by assigning varying degrees of membership to both categories(Akintola et al., 2024). Additionally, it aggregates multiple pollutants and considers their toxicity or benefits, providing a comprehensive risk assessment rather than evaluating each pollutant in isolation. This approach strengthens the reliability of environmental assessments.The focus of this study is to gain knowledge on using the methodology of fuzzy logic-based model as against the conventional method for modeling naturally occurring radionuclides and potentially toxic elements distributed across some major quarry sites in Ogun State, Nigeria as again.

## Materials and methods

### Study area

**Fig. 1:**
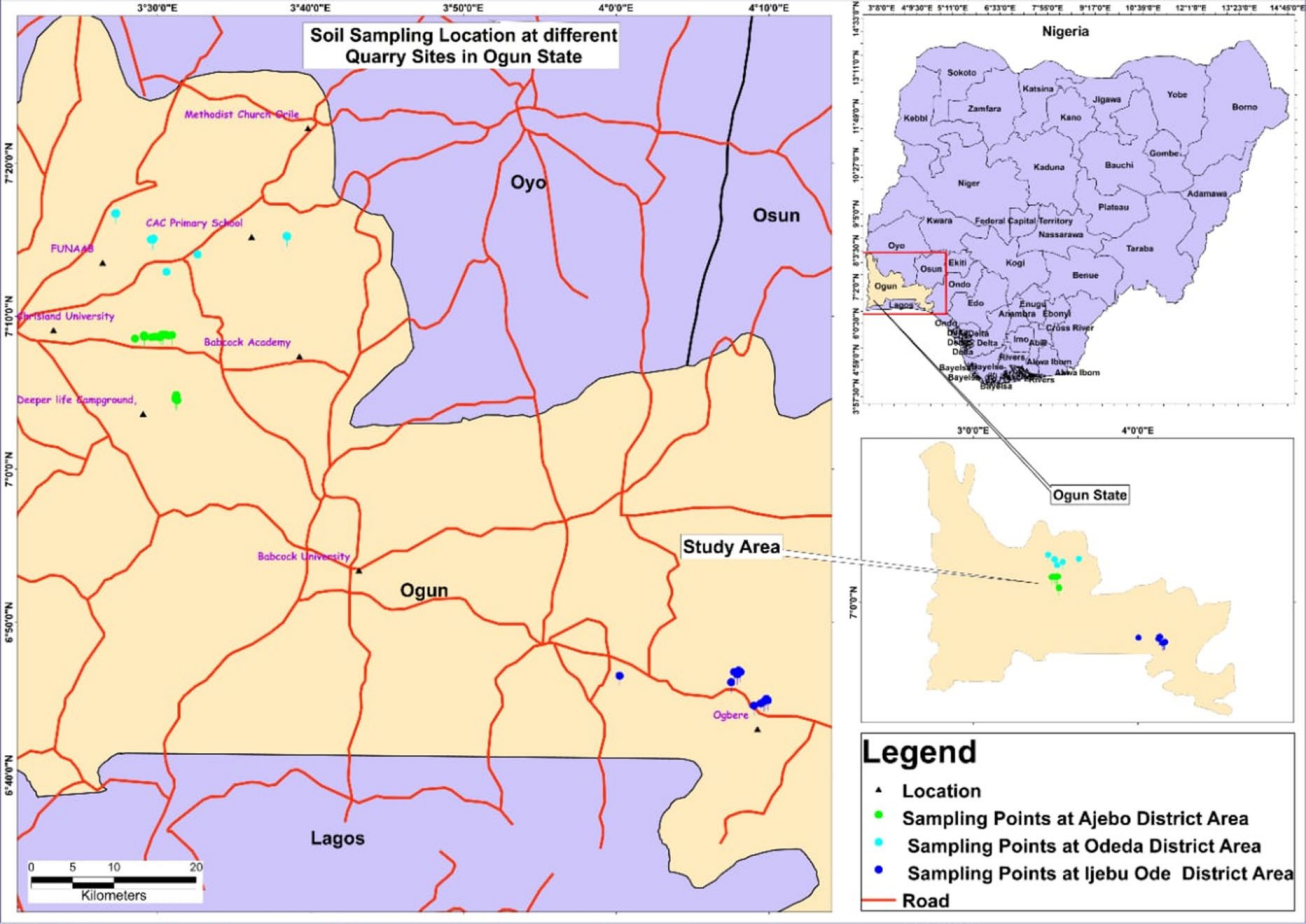
Map showing the sampling points in the quarry sites in all district areas in Ogun State

The study was conducted in Ogun state, located in southwest Nigeria, with a population of over 3.75 million (Olukanni et al., 2020), and had undergone considerable industrialization since its creation in 1976. It has a land mass area of 16,980.55 km^2^ with a density of 220/km^2^ and a coordinate of 6.9980° N, 3.4737° E (Olukanni et al., 2020). It shared a boundary with Oyo State in the northern part, a border with Lagos State and the Atlantic Ocean in the southern part, Ondo State in the east, and the Republic of Benin in the western part of the state. A total of ten (10) selected quarry sites approximately 1.5–7 km apart from each other were selected for the study. The soil samples collected from all the sites were coded to prevent identification errors (Fig. 1). The quarry sites are Skelly(SKY), S and D (S&D), Alabata (ALA), Shogor (SHG), Ratcon (RAT), Safe (SAF), Abl (ABL), Labstar (LAB), 24 Hrs (24H) and Hoy (HOY).

### Sampling and sample preparation

A total of two hundred and fifty (250) soil samples of 1 kg each were collected at ten (10) selected quarry sites in the three (3) senatorial districts in Ogun State. Using a soil auger, the soils were sampled at a depth of 0–15 cm and packed in polyethylene bags along with one control sample each (site with no anthropogenic and lithogenic input from the quarry sites). The control representing each district area was collected to compare the concentrations of the pollutants in the mining site(s) to the living area. The samples were transported to the lab so they could be prepared. Using coning and quartering procedures, twenty-five (25) samples that were obtained from each quarry site were composited in the laboratory for appropriate homogenization, yielding five representative samples that were appropriately labeled. After being sun-dried, ground into a powder, and sieved through a 0.2 mm mesh screen to exclude lumps, pebbles, and organic debris, the representative samples were stored in the lab for further analysis. Personal protective equipment was worn for proper protection during the sampling process.

### Digestion of potential toxic elements

The total potential toxic element present in the sample was assessed using the digestion method.

1 g of the sample was digested using aqua regia (HCl/HNO_3_ ratio 3:1) for 1 h at 110 °C until a transparent solution was obtained. The solutions were filtered through Whatman No. 42 filter paper and diluted to 25 mL with deionized water. Digestion was duplicated to determine the method’s precision, and spiked samples were carried out to recover the PTEs. PTEs concentrations in the soil samples were determined using a thermo scientific inductively coupled plasma optical emission spectrophotometer (ICP-OES) model no ICAP-6500DUO.

### Radioactivity measurement and counting

Using a gamma ray spectrophotometer (GS-2000-Pro) with an Australia detector NaI (Tl) 3 × 3-inch, princeton gamma tech (USA) on the soil samples, the National Institute of Radiation Protection and Research (NIRPR), University of Ibadan, Nigeria, carried out the experiments to measure the soil samples’ radioactivity. The typical soil samples were dried for the entire night at 105 °C to eliminate any remaining moisture. After the material was weighed, 500 g of homogenized material was placed into a cylinder-shaped plastic container measuring 7 cm in height and 6 cm in diameter.

Before gamma testing, the containers were hermetically sealed with adhesive tape for 30 days to allow for secular radioactive equilibrium between 238U and its short-lived progenies. (AERB, 2003; Gbadamosi et al., 2018). An array of potential isotopes and gamma energies were matched by the computer application Maestro window, which was linked to the detector. On all four sides and on top, a lead layer 10 cm thick and 15 cm thick protected the detector. The energy resolution of 2.0 keV and the relative efficiency of 33% at 1.33 MeV were achieved within the system utilizing a 10,800-s counting time. IAEA 315, the International Atomic Energy Agency, provided standard sources for the calibration (IAEA, 2003). Measurements were conducted with reference and soil samples considering background noise. The activity concentrations of ^40^ K, ^232^Th, and ^238^U were discovered by ongoing spectrum research (Jibiri et al., 2014). The peak values for ^40^ K, ^238^U, and ^232^Th are 1460 keV (^40^ K), 1764.5 keV (Bi-214), and 2614.5 keV (^208^Ti), respectively, in order to calculate the activity levels (Bqkg^−1^). According to Jibiri et al. (2014) and Gbadamosi et al. (2018), Eq. 1 was used to calculate the activity concentration (Cs) of the radionuclide after deducting the decay adjustment.

1 $$Sc = \frac{Qnet}{{\varepsilon \gamma Y\gamma Sm Ft}}$$

where: S_c_ = Sample activity concentration, Q_net_ = Net peak area of a peak at energy, F_t_ = Total counting time, Yγ = The yield of the gamma-ray at a particular energy. S_m_ = Sample mass.

$\varepsilon$ γ = Efficiency of the detector for a γ-energy of interest.

The minimum detectable activity (MDA) of each of the radionuclides was obtained for the same counting time as was done for the soil samples, estimated as 0.037 BqKg^−1^ for ^238^U, 0.007 BqKg^−1^ for ^232^Th and 0.18 BqKg^−1^ for ^40^ K.

2 $${\text{LLD }} = { 4}.{65}\left[ {{\raise0.7ex\hbox{${C_{b} }$} \!\mathord{\left/ {\vphantom {{C_{b} } {t_{b} }}}\right.\kern-0pt} \!\lower0.7ex\hbox{${t_{b} }$}}} \right]^{{2}} \times f$$

The background counting time (tb) is measured in seconds, the net background count in the subsequent peak is represented by Cb, and the factor f is used to convert counts per second (cps) to activity concentration (Bq kg-1). Utilizing the known radionuclide activity ^40^ K (578.4 keV), ^238^U (20.9 keV), and ^232^Th (10.47 keV) as a reference standard source allowed for additional calibration of the detector's efficiency. 17.3 Bqkg-1, 5.1 Bqkg-1, and 5.0 Bqkg-1 were the corresponding detection limits for ^40^ K, ^226^Ra, and ^232^Th (Jegede et al., 2019).

### Fuzzy comprehensive assessment of potentially toxic elements and radionuclide in soil

Fuzzy synthetic evaluation (FSE) is a class of fuzzy logic mapping of data that focuses mainly on variable, uncertain, linguistic, and vague information or data. According to Agunbiade et al. (2012), pattern recognition is frequently used to extract logical, trustworthy, systematic, and transparent information from such data collecting for real-world uses. The FSE method’s subdivision, fuzzy comprehensive assessment (FCA) (Shen et al., 2005), uses the matrix created when field data is converted to a fuzzy membership function and then applies the weighting average fuzzy reasoning technique. PTEs and radionuclides were evaluated in this investigation using a variety of FCA procedures, including:

*Step 1*: Selection of parameters and establishment of criteria for assessment.

Parameters that are important and could accurately and reliably assess the contamination level and effectively represent the local environment were selected. A set of functions were assigned to the parameters.

3 $$\theta = \left\{ {\theta_{1} ,\theta_{2} ,\theta_{3} \ldots \theta_{n} } \right\}$$

${\theta }_{1},{\theta }_{2},{\theta }_{3}\dots {\theta }_{n}$ are the parameters to be assessed for this study (PTEs and naturally occurring radionuclide materials), while the ‘*n*’ is the total number of parameters investigated [*n* = 10 (PTEs) and 3(radionuclides) for this study].

Also, regulatory standards were used to establish criteria or limits for each PTEs or radionuclide (FEPA, 1991; USEPA, 1993; UNSCEAR, 2000) corresponding to the membership function. The background concentration of some pollutants that are natural to the environment under study was also considered. (Table 1)

4 $$f = \left( {f_{1} ,f_{2} ,f_{n} } \right)$$

f_1_.f_2–––-_fn are classifications of the parameters, where ‘*n*’ is the total number of classifications for both HMs and NORMs.

**Table 1:** Membership function limits for potentially toxic elements and naturally occurring radionuclide materials

PTEs/NORMs	The parameter limits for the membership function classes		
	*f*_1_	*f*_2_	*f*_3_
As (mg/kg)	0.5	1.0	3.0
Cd (mg/kg)	0.5	1.0	6.0
Co (mg/kg)	1.0	10.0	20.0
Cr (mg/kg)	1.0	5.0	10.0
Cu (mg/kg)	20.0	40.0	90.0
Fe (mg/kg)	50.0	200.0	600.0
Mn (mg/kg)	50.0	100.0	600.0
Ni (mg/kg)	1.0	2.0	10.0
Pb (mg/kg)	1.0	5.0	20.0
Zn (mg/kg)	20.0	40.0	90.0
^40^ K (Bq/kg)	200.0	400.0	900.0
^238^U (Bq/kg)	15.0	30.0	80.0
^232^Th (Bq/kg)	20.0	40.0	90.0

The *f*_1_, *f*_2_, and *f*_3_ classification corresponds to pristine, moderately enriched, and extremely impacted, respectively

*Step 2:* Membership function formulation.

The soil quality assessment was classified into 3 levels. These classifications were based on the degree of pollution impact on the site under investigation. The classification was based on classification I—Pristine condition; Classification II – Moderately enriched condition; Classification III – Extremely impacted condition for potentially toxic elements. The classification I, II, and III correspond to limit *f*_1_, *f*_2_, and *f*_3_, respectively. For naturally occurring radionuclide materials, classification was based on Classification I – Low impact condition, Classification II – Medium impact condition, and Classification III – High impact condition. The classification I, II, and III correspond to limit *f*_1_, *f*_2_ and *f*_3_ respectively. The concentration of the parameters (PTEs and radionuclide) was used in relation to these limits to formulate fuzzy membership function based on the expressions below (Eqs. 5 – 7).

5 $$\theta _{1} \left( y \right) = \left( {\begin{array}{*{20}l} 1 \hfill &\quad {if~y~ \le f_{1} } \hfill \\ {\left( {\frac{{f_{2} - y}}{{f_{2} - f_{1} }}} \right)} \hfill &\quad {~if~f_{1} < y < f_{2} } \hfill \\ 0 \hfill &\quad {~if~y~ \ge f_{2} } \hfill \\ \end{array} } \right)$$

6 $$\theta _{2} \left( y \right) = \left( {\begin{array}{*{20}l} 0 \hfill &\quad {~if~y~ \le f_{1} } \hfill \\ {1 - f_{1} \left( y \right)} \hfill &\quad {~if~f_{1} < y \le f_{2} } \hfill \\ {\frac{{\left( {f_{3} - y} \right)}}{{\left( {f_{3} - f_{2} } \right)}}} \hfill &\quad {if~f_{2} < y < f_{3} } \hfill \\ 0 \hfill &\quad {~if~y~ \ge f_{3} } \hfill \\ \end{array} } \right)$$

7 $$\theta _{3} \left( y \right) = \left( {\begin{array}{*{20}l} 0 \hfill &\quad {if~y~ \le f_{2} } \hfill \\ {1 - f_{2} \left( y \right)} \hfill &\quad {if~f_{2} < y < f_{3} } \hfill \\ 1 \hfill &\quad {~if~y~ \ge ~f_{3} } \hfill \\ \end{array} } \right)$$

where y is the observed data from chemical analysis of the PTEs and NORMs used in the assessment ${\theta }_{1}\left(y\right)$,${\theta }_{2}\left(y\right)$, ( ) θ3y are the fuzzy membership functions corresponding to I, II, and III, respectively.

*Step 3* – The calculation of membership function and matrix formulation.

Inserting the observed data at each monitoring point and the restrictions into the expressions allowed the development of the membership functions of the observed monitoring data at each study site in Step 2, which were then arranged into a fuzzy matrix ${X}_{K}$ according to Eq. 8.

8 $$X_{K} = \left( {\begin{array}{*{20}c} {\theta _{{11}} } & {\theta _{{12}} } & \cdots & {\theta _{{1n}} } \\ {\theta _{{21}} } & {\theta _{{22}} } & \cdots & {\theta _{{2n}} } \\ \vdots & \vdots & \vdots & \vdots \\ {\theta _{{n1}} } & {\theta _{{n2}} } & {} & {\theta _{{nm}} } \\ \end{array} } \right)$$

where ${\theta }_{ij}$ is the membership degree of the obtained classification for *j*th number of PTEs and NORMs and *i*th number of limit classification. Therefore, *i* = 1,2…n and *j* = 1,2…m, where m and n is the number of PTEs (10) / radionuclides (3) that was investigated and the number of criteria classification, and ${X}_{K}$ is the fuzzy matrix for site ‘*k*’ under investigation respectively.

*Step 4:* Develop weight matrix.

Fuzzy comprehensive assessments require a consistent weighting scheme for each parameter at each site under investigation. Analytical Hierarchy Process (AHP) provided the basis for weight distribution. Prioritizing options allows it to address decision-making challenges across a range of domains. Equations 9 and 10 provide the mathematical expression for the weight allocation used in this investigation, which was based on the ratio of the observed data contribution for each PTE to the total weight.

9 $$W_{i\left( k \right)} = \frac{{a_{i\left( k \right)} }}{{\mathop \sum \nolimits_{i = 1}^{n} a_{i} \left( k \right)}}$$

10 $$a_{i\left( k \right)} = \frac{{C_{i\left( k \right)} }}{{S_{i} }}$$

where ${C}_{i(k)}$ is the concentration of the PTEs number ith in monitoring site ‘*k*’ and ${S}_{i}$ is the average limits in formulating the membership function for each PTEs and NORMs. The values obtained from ${a}_{i}$ are substituted into equation ${W}_{i(k)}$ of each site’s assessment parameters (PTEs and NORMs). The weight obtained can then be expressed as the weight matrix ${P}_{k}$ (Eq. 11).

11 $$P_{k} = \left( {\begin{array}{*{20}c} {W_{i\left( k \right)} } \\ {W_{2\left( k \right)} } \\ \vdots \\ {W_{m\left( k \right)} } \\ \end{array} } \right)$$

With “*m*” denoting the number of PTEs and radionuclide species under investigation. This method of allocating weight makes sense since it considers the impact of the observed concentration of parameter (*y*) that is significantly higher or lower than the accepted limits when determining the weight for each parameter.

*Step 5*: The Determination of Fuzzy Algorithm.

Equation 8 (the fuzzy matrix) plus Eq. 11 (the weight matrix) were multiplied to determine the fuzzy algorithm. The matrix index, which categorized the sites’ level of contamination into any of the three classifications given, was produced by taking the product moment of the fuzzy matrix and the weight matrix ${P}_{K}$. Equation 12 provides an expression for the matrix’s product.

12 $$X_{K} P_{K} = \left[ {\begin{array}{*{20}c} {k_{1} } \\ {k_{2} } \\ : \\ {k_{J} } \\ \end{array} } \right]$$

### Statistical analysis

The radionuclides and potentially toxic elements found in the soil samples were analyzed using descriptive statistical methods in this work. Using IBM SPSS statistics (version 20, Inc., Chicago, IL), a commercial statistical software tool, the descriptive analysis includes the mean, skewness, kurtosis, maximum, minimum, standard deviation, and coefficient of variation.

### Quality control

The results were validated using quality control. To identify potential contamination in the analytical materials and evaluate the accuracy and bias of the method, recovery studies, duplicates, and reagent blanks were included in the study. The cleanliness of the area where the analyses were conducted had a direct impact on the accuracy and precision of the procedure, and samples were treated with great care. Throughout the study, deionized water was utilized along with high-purity analytical grade reagents. The regression coefficients showed that the calibration curves were linear within the concentration range. (*R*^2^ > 0.9996). The instruments (ICP-OES) detection limits for the elements are Arsenic (2.14 µg/L), Cadmium (0.07 µg/L), Cobalt (0.51 µg/L), Chromium (0.21 µg/L), Copper (0.39 µg/L), Iron (0.25 µg/L), Nickel (0.36 µg/L), Lead (1.06 µg/L), Manganese (0.07 µg/L) and Zinc (0.19 µg/L).

The precision of the analysis for the standard solution was ensured by performing the analysis in triplicate, achieving a precision within 10%. Procedure blanks and quality control samples, developed from standard solutions, were used to ensure sample accuracy. Accuracy was validated using standard reference materials (SRM) purchased from Merck (Darmstadt, Germany). Soil samples from each location were spiked with the standard reference materials for recovery tests using the same extraction process. The recovery rates of potentially toxic metals were as follows: 99.45% for arsenic, 98.20% for cadmium, 99.40% for cobalt, 95.85% for chromium, 99.84% for copper, 101.55% for iron, 98.65% for nickel, 97.82% for lead, 97.86% for manganese, and 98.75% for zinc.

## Results and discussion

### Fuzzy comprehensive assessment modelling

#### Fuzzy comprehensive assessment for potentially toxic elements

The fuzzy matrix was formed using the average values of PTEs concentrations in the soil sample as presented (Tables 2a and b).

**Table 2:** Concentration of potentially toxic elements (*n* = 5) in all the quarry sites using descriptive statistical analysis

Site	As(mg/kg)	Cd(mg/kg)	Co(mg/kg)	Cr(mg/kg)	Cu(mg/kg)
ABL	10.97 ± 1.12	0.45 ± 0.28	7.71 ± 1.34	23.28 ± 2.48	28.98 ± 2.7
SHG	6.98 ± 1.67	0.53 ± 0.08	5.73 ± 1.30	34.07 ± 5.40	28.43 ± 4.31
HOY	15.93 ± 2.40	BDL	13.55 ± 2.33	25.4 ± 4.93	26.15 ± 6.20
24H	5.67 ± 1.53	0.65 ± 0.10	9.37 ± 2.58	21.35 ± 1.63	19.87 ± 1.98
LAB	11.40 ± 2.70	0.87 ± 0.33	16.14 ± 1.22	56.57 ± 2.76	82.43 ± 7.94
SKY	7.57 ± 1.86	1.26 ± 0.6	27.25 ± 1.14	57.07 ± 3.14	23.25 ± 3.98
SAD	7.36 ± 1.69	0.48 ± 0.078	8.97 ± 0.94	23.53 ± 2.60	35.95 ± 5.21
ALA	10.52 ± 1.75	BDL	5.56 ± 1.34	18.68 ± 1.54	23.68 ± 2.86
SAF	5.62 ± 0.85	0.46 ± 0.07	12.63 ± 1.78	61.43 ± 6.33	26.66 ± 2.11
RAT	5.81 ± 0.67	0.39 ± 0.04	6.84 ± 0.61	30.70 ± 4.14	12.40 ± 1.31
Mean** ± **SE	8.54 ± 1.68	0.52 ± 0.14	11.78 ± 1.47	36.53 ± 3.61	30.98 ± 3.99
Kurtosis	1.55	0.53	2.86	− 1.64	6.67
Skewness	1.4	0.47	1.58	0.62	2.44
Range	10.31 ± 2.69	1.26 ± 0.48	21.69 ± 2.54	42.75 ± 1.86	70.03 ± 7.75
Minimum	5.62 ± 0.85	BDL	5.56 ± 1.34	18.68 ± 1.54	12.40 ± 1.31
Maximum	15.93 ± 2.40	1.26 ± 0.60	27.25 ± 1.14	61.43 ± 6.33	82.43 ± 7.94
Site	Fe(mg/kg)	Mn(mg/kg)	Ni(mg/kg)	Pb(mg/kg)	Zn(mg/kg)
ABL	26,902.50 ± 290.75	194.97 ± 14.81	4.96 ± 1.57	6.00 ± 1.89	84.70 ± 4.45
SHG	21,907.50 ± 135.65	289.07 ± 11.24	13.03 ± 4.74	7.13 ± 1.44	42.97 ± 4.13
HOY	32,650.00 ± 499.40	262.70 ± 12.17	5.63 ± 1.99	9.28 ± 1.62	142.00 ± 7.50
24H	15,035.00 ± 81.12	168.97 ± 5.93	6.53 ± 1.50	17.87 ± 2.8	51.27 ± 6.28
LAB	36,520.00 ± 292.20	353.30 ± 20.84	15.23 ± 1.77	4.44 ± 0.80	119.90 ± 8.54
SKY	25,117.50 ± 126.20	248.25 ± 12.48	25.54 ± 2.50	5.66 ± 1.23	63.10 ± 5.61
SAD	21,395.00 ± 109.38	234.95 ± 11.97	13.65 ± 1.73	4.73 ± 0.91	56.73 ± 2.30
ALA	23,177.50 ± 86.96	302.05 ± 7.53	7.68 ± 1.21	5.65 ± 1.65	49.93 ± 1.57
SAF	21,085.00 ± 78.05	291.78 ± 6.02	22.47 ± 1.77	8.21 ± 0.97	61.70 ± 5.89
RAT	21,197.50 ± 41.78	236.30 ± 14.49	9.58 ± 1.41	11.55 ± 1.33	44.93 ± 6.83
Mean** ± **SE	24,231.67 ± 161.20	265.26 ± 11.41	13.26 ± 2.10	8.281 ± 1.40	70.28 ± 5.41
Kurtosis	0.63	0.9	− 0.41	2.69	1.14
Skewness	0.91	-0.21	0.79	1.6	1.56
Range	21,485.00 ± 279.36	184.33 ± 16.78	19.91 ± 3.78	13.43 ± 1.68	99.03 ± 4.87
Minimum	15,035.00 ± 81.12	168.97 ± 5.93	5.63 ± 1.99	4.44 ± 0.80	42.97 ± 4.13
Maximum	36,520.00 ± 292.20	353.30 ± 20.84	25.54 ± 2.50	17.87 ± 2.80	142.00 ± 7.50

*BDL* below detection limit, *n* number of samples, *SE* standard error

**Scheme 1:**
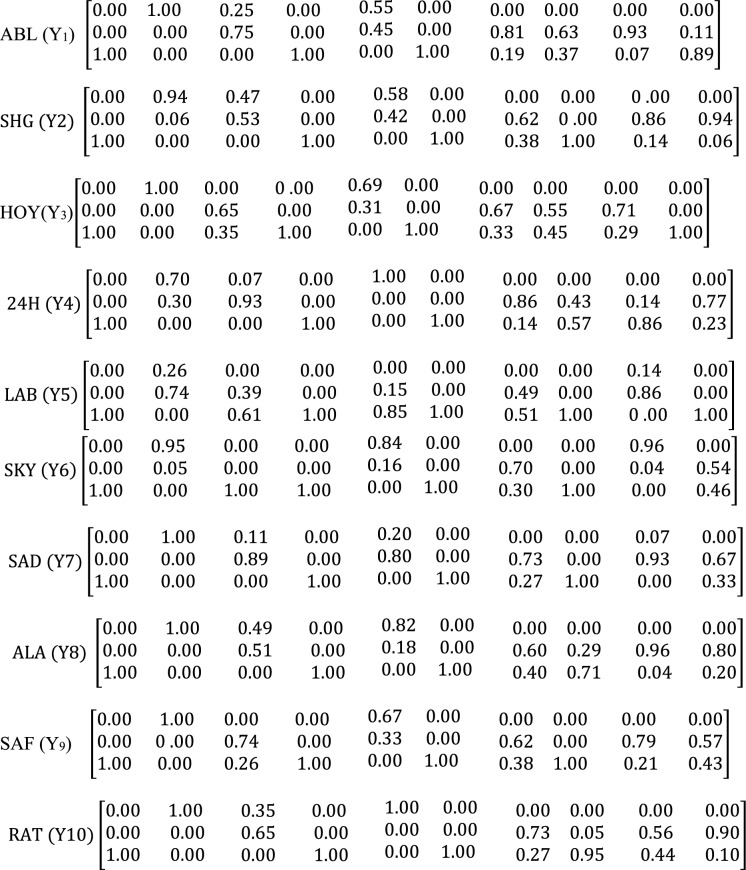
Fuzzy membership matrix Y_k_ for the ten (10) sampling sites and ten (10) Potentially Toxic Elements in the soil samples. Each row of the Y_k_ represents As, Cd, Co, Cr, Cu, Fe, Mn, Ni, Pb, and Zn

The data presented in Tables 2a and 2b were modeled with a fuzzy comprehensive assessment, which used mapping and function principles to study the degree of contamination level and impact of activities. Different fuzzy matrix for the soil sample metals at quarry sites ABL, SHG, HOY, 24H, LAB, SKY, SAD, ALA, SAF and RAT are presented as *Y*_1_, *Y*_2_, *Y*_3_, *Y*_4_, *Y*_5_, *Y*6, *Y*_7_, *Y*_8_, *Y*_9_, *Y*_10_ respectively and their weight matrices correspondingly are *P*_1_, *P*_2_, *P*_3_, *P*_4_, *P*_5_, *P*_6_, *P*_7_, *P*_8_, *P*_9_, *P*_10_. The PTEs trend used in the fuzzy matrix system is Arsenic, Cadmium, Cobalt, Chromium, Copper, Iron, Manganese, Nickel, Lead, and Zinc. The classification of the PTEs was done in three ways—pristine, moderately enriched, and extremely impacted. According to the fuzzy matrices, the major contaminants present in the sites were Fe and As. These elements had the highest membership in the extremely impacted class (100%) in all sites. While Fe may be considered less toxic, the presence of As in the extremely impacted class is a call for concern. Ni also revealed a high membership in the extremely impacted class for 8 sites (57 – 100%). As, Ni and Cr have been toxic elements of international concern and have been classified by USEPA as a priority pollutant because of their potential carcinogenicity (Agunbiade et al., 2012). In the moderately enriched class, the toxic elements were Pb (14 – 96%), Zn (11 – 94%), Co (39 – 93%), and Mn (49 – 81%) in seven (7), seven (7), nine (9) and ten (10) sites respectively. Some metals were found in the pristine class and thus not classified as pollutants in the environment under investigation. Cd and Cu have a high membership in the pristine class in all the sites, and eight (8) sites, respectively, but Cu was moderately impacted and extremely impacted in SAD and LAB sites, respectively (Scheme 1).

**Scheme 2:**
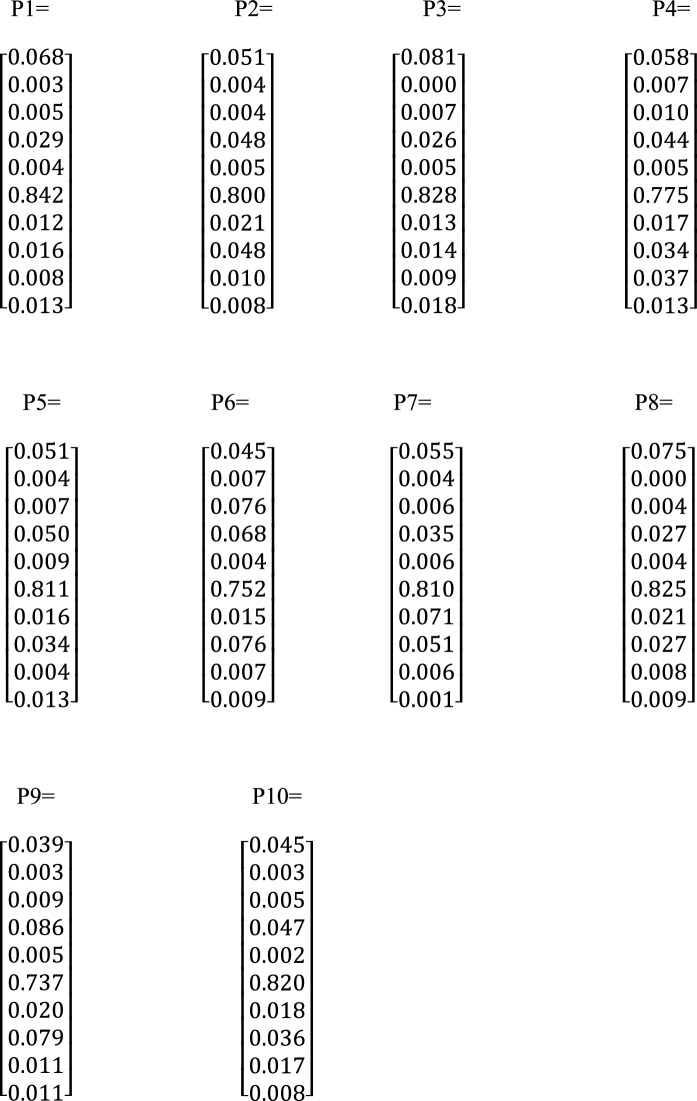
Weight matrix P_k_ for the ten (10) sampling sites and ten (10) Potentially Toxic Elements in the soil samples. Each row of the P_k_ represents As, Cd, Co, Cr, Cu, Fe, Mn, Ni, Pb, and Zn, respectively

The weight matrices (Pi) were calculated (Scheme 2). It was discovered that Fe was the main contaminant in all the sites, with a percentage ranging from 73.7 – 84.2%, with SAF and ABL sites having the lowest and highest sites, respectively. As and Cr also had higher percentages ranging from 3.9 – 8.1% and 2.6 – 8.6%, respectively, with SAF and HOY sites having the lowest and highest percentage for As and HOY and SAF sites having the lowest and highest percentage for Cr. Cadmium contributed the lowest percentage of PTEs in all the sites except in 24H, where Cu was the lowest (0.05%). This suggests an environment free of pollution and diseases that might arise from Cd. Copper was the next least contributor to the sites (0.2–0.9%). These results revealed that Fe and As are metals of focus for management, while Cadmium and copper are used to control the environment. Geologic processes mainly cause arsenic poisoning in the environment, but it can also be exacerbated by anthropogenic activities, such as using pesticides that contain Arsenic and mining and smelting (Banerji et al., 2018). Studies have been done on soil and the connections between bioaccessibility and the chemical forms of As. It is widely known that crystalline and amorphous Fe oxide has strong binding properties for As in soils. It is crucial for As retention because soil-based Fe oxide can co-precipitate with Arsenic or adsorbed on it (Jeong et al., 2017). The fuzzy logic weighted average calculated for the PTEs under investigation in all sites has properly classified the sites into different degrees of contamination. The pristine class was 0.3 – 1.8%, with LAB and HOY sites having the lowest percentage and ABL sites having the highest percentage. This showed that the ABL site was cleaner and free of pollution than other sites. Furthermore, the moderately enriched class ranges between 1.8 – 9.4%, with LAB and SAD sites having the lowest and highest percentages, respectively. This was suggestive that the SAD site was more moderately enriched than any.

Other sites are under investigation. The extremely impacted class ranges between 93.3 – 97.9%, with 24H and LAB sites having the lowest and highest percentage, respectively.

**Scheme 3:**
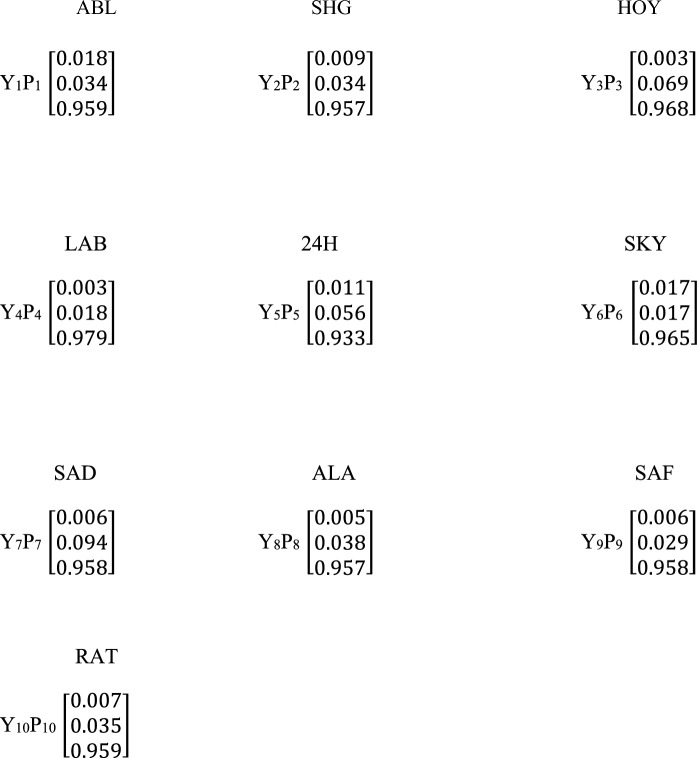
Fuzzy Logic weighted average for the Potentially Toxic Elements

The LAB site was the most contaminated site due to the accumulation/ combined effect of all the metals present (Scheme 3). A high percentage of membership in the extremely impacted class is because of a high concentration of Fe present in all the sites under study. A high concentration of Fe can be linked to using machines, such as crushers, drillers, and wheel loaders, that are produced from iron for crushing stones and rocks in the quarry sites. In comparison to other significant elements that make up about 5% of the lithosphere, iron is the fourth most prevalent element in soil, with a prevalence of between 0.5 and 5%. (Rout & Sahoo, 2015; Tavakoli et al., 2014). In addition to being a necessary mineral, iron is crucial to many biological functions. According to De Mello Gabriel et al., in (2021), this element is relatively abundant worldwide (about 45%), with concentrations varying greatly depending on the soil type and other sources’ presence. Soil contains the most significant iron in the two oxidation states of ferrous and ferric, respectively, Fe(II) and Fe(III) (Colombo et al. (2014). Due to the redox equilibrium, which depends on pH, temperature, and other factors in a solution, these oxidation states are easily convertible from one to another. According to Hoffmann (2005) and Mello Gabriel et al., 2021, ferrous and ferric iron are both engaged in the creation of colloids, particulate phases, and organic and inorganic soluble complexes. For the carbon and nutrient cycles in flooded soils, the transition between ferric and ferrous iron is crucial. In the iron reduction reaction, which is driven by microorganisms, labile organic matter is known to operate as an electron donor (Emsens et al., 2016; Gabriel et al., 2020). The iron reduction is related to the suppression of methanogenesis. It is characteristic of mobility that most of the iron in flooded soils is in the ferrous state (Gabriel et al., 2020; Mello Gabriel et al., 2021). Also, due to the geological structure of the environment in this part of the world (Nigeria), it is evident that a high amount of Fe is usually found in the earth's crust, which has been linked to natural activities (Agunbiade et al., 2012). It is therefore revealed from the results that the soil samples in the quarry sites reported in this research are impacted by PTEs that are discharged or released from natural and anthropogenic activities and possibly have accumulated over a long period, thus causing pollution in the environment under investigation.

#### Fuzzy comprehensive assessment for naturally occurring radionuclide materials

**Scheme 4:**
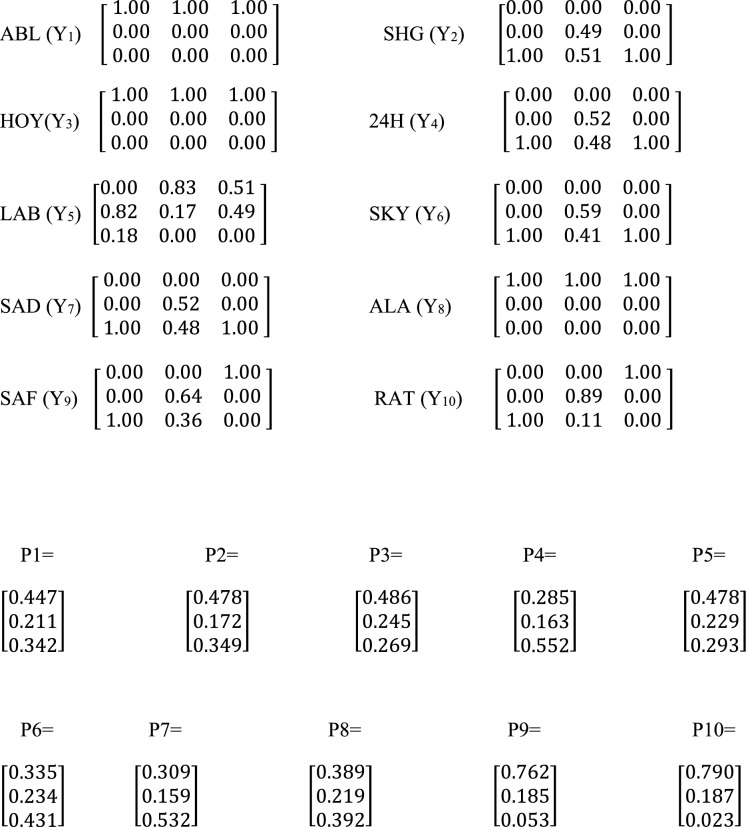
Fuzzy comprehensive assessment membership matrix Y_k_ and corresponding weight matrix Px for the ten (10) sampling sites and three (3) radionuclides in the soil samples. Each column and row of the Y_k_ and P_k_ represent ^40^ K, ^238^U, ^and 232^Th respectively

The fuzzy matrix was formed using the average values of the activity concentrations of NORMs in the soil sample (Table 3). The fuzzy membership function obtained for the naturally occurring radionuclide materials (NORMs) in the order ^40^ K, ^238^U and ^232^Th at the ten sites (SHG SKY, SAD, ALA, 24H, ABL, HOY, LAB, SAF and RAT) are represented by fuzzy matrix (X_k_) X_1_, X_2_, X_3_, X_4_, X_5_, X6, X_7_, X_8_, X_9_, X_10_ (Scheme 4), and their weight matrix correspondingly are *P*_1_, *P*_2_, *P*_3_, *P*_4_, *P*_5_, *P*_6_, *P*_7_, *P*_8_, *P*_9_, *P*_10_. Naturally occurring radionuclide materials (NORMs) are classified into pristine, moderately enriched, and extremely impacted. The membership matrix of the NORMs confirmed them to be between pristine and extremely impacted. The major contaminant present in the site based on the fuzzy matrices derived from the membership is ^40^ K (in six sites). These NORMs (^40^ K) have a high percentage classification in the high-impact membership with a significant percentage in the pristine classification. Uranium contributed more in the moderately enriched membership (6 sites), with a percentage ranging between 49 – 89%, while it belongs to pristine membership in the remaining four (4) sites under investigation. However, ^232^Th was found in the pristine class, and it was suggestive to be safe in the quarry sites. It has a high membership in the pristine class in six (6) sites, with a significant amount of membership in the extremely impacted class (4 sites).

**Table 3:** Activity concentration (n = 5) of radionuclides in the soil sample using descriptive statistics

Site	K-40 (Bq/kg)	U-238 (Bq/kg)	Th-232 (Bq/kg)
SHG	2056.44 ± 359.42	55.42 ± 24.88	150.10 ± 73.45
SKY	967.04 ± 231.50	50.54 ± 13.72	124.10 ± 51.0
SAD	1046.26 ± 386.54	40.27 ± 11.35	179.70 ± 95.62
ALA	76.78 ± 44.76	3.24 ± 1.82	7.71 ± 5.53
24H	1262.48 ± 922	53.97 ± 12.55	244.36 ± 89.84
ABL	145.02 ± 66.77	5.13 ± 1.28	11.10 ± 7.40
HOY	94.67 ± 24.20	3.57 ± 1.90	5.24 ± 3.90
LAB	488.05 ± 110.73	17.52 ± 2.00	29.83 ± 30.21
SAF	2647.88 ± 179.44	48.21 ± 18.96	18.61 ± 8.02
RAT	2552.97 ± 137.18	45.48 ± 10.0	7.38 ± 2.57
Mean** ± **SE	1133.76 ± 498.87	32.34 ± 19.67	77.81 ± 37.23
Kurtosis	− 1.24	− 1.93	− 0.74
Skewness	0.51	− 0.46	0.89
Range	2571.10 ± 473.27	52.18 ± 3.70	239.12 ± 47.62
Minimum	76.78 ± 44.76	3.24 ± 1.82	5.24 ± 3.90
Maximum	2647.88 ± 179.44	55.42 ± 24.88	244.36 ± 89.84

*BDL* below detection limit, *n* number of samples, *SE* standard error

**Scheme 5:**
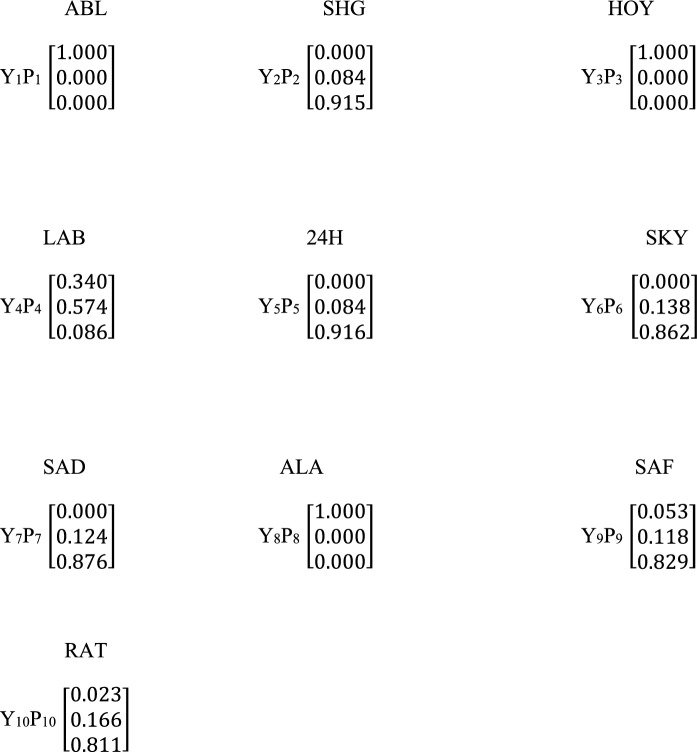
Fuzzy comprehensive assessment weighted average for the naturally occurring radionuclide materials

The weight matrices of the NORMs were calculated according to Eq. (11) and shown in scheme 4. It was evident from the result that ^40^ K was the main contributor at ABL (44.7%), SHG (47.8%), LAB (47.8%), HOY (48.6%), SAF (76.2%) and RAT (79.0%) sites. Also, ^232^Th was the highest contributor of naturally occurring radionuclide materials at ALA (39.2%), SKY (43.1%), SAD (53.2%), and 24H (55.2%) sites. The NORMs that contributed the least to the pollution level in all the sites was ^238^U, which showed contribution levels of 17.2%, 15,9%, 23.4%, 21.9%, 16.3%, 21.1%, 24.5% and 22.9% in SHG, SAD, SKY, ALA, 24H, ABL, HOY, and LAB sites respectively. The sites were classified adequately into different degrees of contamination using the calculated Fuzzy Logic weighted average (Scheme 5). The pristine class was in the range (0–100%), moderately enriched (0 – 57.4%) and extremely impacted (0 – 91.6%). The results showed that ALA, HOY, and ABL sites are 100% clean and free from pollution arising from naturally occurring radionuclide materials. Furthermore, the LAB site was moderately polluted due to its contribution (57.4%) in the classification range. 24H, SHG, SAD, SKY, SAF, and RAT sites are highly impacted by NORMs pollutants and are considered polluted sites due to a high percentage of NORMs by a combined effect. (81.1 – 91.6%). Six sites were confirmed to be highly polluted, while 3 sites had a clean environment due to low impact (pristine) membership, which they fell into in the classification criteria.

## Conclusion

This study focused on the distribution of potentially toxic elements (PTEs) and naturally occurring radionuclide materials in the quarry soils. The fuzzy logic weighted average calculated for the PTEs under investigation in all sites has adequately classified the sites into different degrees of contamination. ABL site has the highest percentage in the pristine class, which shows that the site was cleaner and less polluted than other sites. SAD sites have the highest percentage in the moderately enriched class than other sites under investigation. In contrast, the LAB site was found in the extremely impacted class, thus confirming it to be the most contaminated site due to the combined effect of all the PTEs being investigated. A high percentage of membership in the extremely impacted class was because of a high concentration of Fe present in all the sites under study. A high concentration of Fe can be linked to machines such as crushers, drillers, and wheel loaders produced from iron for crushing stones and rocks in the quarry sites. Also, due to the geological structure of the environment in this part of the world (Nigeria), it was evident that a high amount of Fe was usually found in the earth’s crust, which has been linked to natural activities. The simple fuzzy logic classification (SFC) showed that many sites are polluted with PTEs, mainly due to the natural contributions of Fe and Mn present in high concentrations. A high concentration of Fe and Mn has become a trend in most of the Nigeria soils. The SFC also confirmed As, Fe, Zn, and Ni to be priority metals to be controlled in the quarry sites. ^40^ K and ^238^U have high-impact membership in all the quarry sites and thus should be appropriately monitored. Six sites were found to be highly polluted with NORMs using SFC.

To address the significant pollution identified in this study, strict pollution control measures should be implemented at quarry sites, with particular attention to the LAB site, which is classified as extremely impacted. Regular maintenance of quarry equipment, such as crushers and drillers, is essential to minimize the release of Fe and other potentially toxic elements (PTEs). Monitoring protocols should prioritize metals like As, Fe, Zn, and Ni, while radionuclides such as ^40^ K and ^238^U require consistent oversight to mitigate their environmental and health risks. Additionally, region-specific environmental regulations should be developed to address both natural and anthropogenic contributions to quarry pollution, considering the unique geological conditions of Nigeria.

Future research should evaluate the long-term impacts of PTEs and NORMs on ecosystems and public health and explore remediation techniques such as phytoremediation or soil stabilization. Collaboration with stakeholders, including government agencies and local communities, is critical to establishing effective guidelines and promoting sustainable quarrying practices. Educational initiatives should raise awareness among operators and residents about the risks of quarry pollution and the importance of adhering to regulatory standards. These combined efforts can help mitigate contamination, safeguard human health, and promote sustainable development in quarry regions.

## Data Availability

No datasets were generated or analysed during the current study.
